# Stepwise complexometric determination of aluminium, titanium and iron concentrations in silica sand and allied materials

**DOI:** 10.1186/1752-153X-1-24

**Published:** 2007-10-19

**Authors:** Nijhuma Kayal, Nahar Singh

**Affiliations:** 1 Analytical Chemistry section, Central Glass and Ceramic Research Institute, Kolkata-32, India; 2 Analytical Chemistry section, National Physical laboratory, Dr K.S. Krishnan Marg, New Delhi-12, India

## Abstract

**Background:**

This study aimed at measuring the quantities of Al, Ti and Fe in silica sand and allied materials employing a complexometric method in the same analyte and a stepwise indirect titration with EDTA. The method involves the complexation of Al, Ti and Fe with excess EDTA and the selective de-complexation of TiO-EDTA and Al-EDTA complexes with tartaric acid and NaF respectively. In addition to its simplicity, rapidity and accuracy, the proposed method does not require the use of a separation technique or any sophisticated instrumentation.

**Results:**

Each of the test samples were analyzed five times using the proposed method. The method's accuracy was confirmed by analyzing the US National Institute of Standards and Technology's (NIST) Standard Reference Materials (SRM) 81a, 89 and IPT SRM 61 using the procedure proposed, in addition to analyzing Ti and Fe levels by spectrophotometry and that of Al by complexometry.

**Conclusion:**

The study shows that there is good agreement between the proposed and existing methods. The standard deviations of the measurements were calculated by analyzing five replicates of each sample, and were found to be less than 1.5% in our method.

## 1. Background

Silica sand is a white or colourless crystalline compound, occurringabundantly as quartz, sand, flint, agate and in many minerals [1]. It is used as an essential raw material in the production of glass, foundry, abrasives, filters, ceramics, in chemical and hydraulic fracturing and on oil fields [2]. Silica sand contains Al, Fe, Ti, Ca, Mg, Mn, Na and K as impurities in varying quantities. Iron, aluminium and titanium impurities can alter the colour, chemical, optical and mechanical properties of the product [3,4].

In common glass and allied materials, Al is associated with moderate to low quantities of Ti and Fe. For such materials, the quantities of these metals are determined separately by complexometric and colorimetric methods [5,6]. In the direct titrations however, Al, Ti and Fe interact with one another. In such instances, the concentration of the constituents is determined after separating the interacting elements using a suitable precipitating or complexing agent [7,8]. Pribil and Vesely [9] devised a method for the complexometric determination of Ti, Fe and Al when present together. Ti is separated as the hydroxide in the presence of triethanolamine, which also masks the Fe and Al in the filtrate. The quantities of Fe and Al are determined by complexometry, while that of Ti is determined by spectrophotometry after Ti(OH)_4 _has dissolved. This method has the disadvantage that Fe is also co-precipitated when present in large amounts.

Recently several instrumental techniques such as inductively coupled plasma (ICP) emission and atomic absorption spectrometry (AAS) in such matrixes, have been adopted for the determination of the trace metallic impurities [10-14]. Voinvitch et al. [15] have determined Al in the presence of Ti and Fe by employing an excess of EDTA with ZnCl_2 _as the back titrant at pH 5–6 (in presence of tartaric acid, diammmonium phosphate and fluoride) and dithiozone indicator. In using this method it has been observed that the end point can be affected by the reaction of tartaric acid with Zn as well as dithiozone indicator's slight ability to absorb titanium phosphate.

### New proposed method

Therefore the measurement of Al, Ti and Fe quantities using complexometric methods presents a significant challenge. Having borne in mind the earlier problems, a new approach has been employed in which zinc chloride is replaced with lead nitrate as the back titrant. In the proposed method the quantities of aluminium, titanium and iron present in the same analyte are determined followed by stepwise indirect titration with EDTA, without any seperation steps. In the first step, all three elements are complexed with an excess of EDTA. In the second step, the TiO-EDTA and Al-EDTA complexes are decomplexed selectively and quantitatively by using tartaric acid and NaF respectively. Finally the released EDTA is back titrated with lead nitrate. Tartaric acid was chosen because it does not react with lead nitrate, as in case of using zinc chloride.

A variety of silica sand and allied materials of different origins was analyzed for Al, Fe and Ti using the proposed method, which was deemed very simple, reliable and rapid. The method also allows the determination of trace amount of these metallic species. The results obtained for NIST SRM 81a, 89 and IPT SRM 61 are in good agreement with certified results.

## 2. Results and discussion

In the complexometric determination of Al, Ti and Fe in silica sand and allied materials using stepwise indirect titration with EDTA, the aim is to release EDTA step by step from the mixture of Al-EDTA, TiO-EDTA and Fe-EDTA. The addition of sodium fluoride releases both aluminium and titanium. Therefore tartaric acid is added before NaF, which selectively de-complexes titanium. It has been observed that EDTA released on the addition of tartaric acid should be titrated under hot conditions, because at room temperature EDTA is released partially and poor results are obtained. Therefore it can be concluded that the method is strongly dependent upon temperature and pH, with the optimum temperature and pH for the titration of Ti being in the range of 60–70°C and pH 5.2 to 5.5 respectively. Table 1 shows the results for Al, Ti, and Fe in synthetic solution. It can be seen that in cases of higher Ti and Fe concentrations (above 6%) the proposed method is not suitable, whilst at lower concentrations (less than 0.01%) more uncertainty is observed. Hence it can be concluded that the method is applicable over the concentration range 6% to 0.015%.

**Table 1:** Determination of Al, Fe and Ti quantities in synthetic solution

Al taken/mg	Al found/mg	Deviation/mg	Fe taken/mg	Fe found/mg	Deviation/mg	Ti taken/mg	Ti found/mg	Deviation/mg
20	19.95	-0.05	7.0	6.82	-0.18	7.5	6.67	-0.83
15.6	15.60	_	4.45	4.41	-0.04	4.5	4.46	-0.04
10.4	10.37	-0.03	3.2	3.22	+0.02	3.0	3.02	+0.02
5.75	5.78	+0.03	1.0	1.0	_	1.55	1.5	-0.05
3.20	3.20	_	0.5	0.47	-0.03	0.8	0.84	+0.04
1.50	1.49	-0.01	0.01	0.018	+0.008	0.01	0.015	+0.05

The proposed method has been applied to five silica sand samples and three-reference materials. The analytical results for the five test samples of silica sand and allied materials are given in Tables 2, 3, 4, 5, 6, while the 'accuracy check' results for the standard reference materials NIST SRM 81a glass sand, SRM 89 and IPT SRM No.61 are given in Tables 7, 8, 9. In order to check the reliability of the method, the concentration of iron and titanium were also determined by colorimetry [6] and that of aluminium by complexometry [5]. Tables 2, 3, 4, 5, 6, 7, 8, 9 reveal that the results obtained from the existing and proposed methods are in good agreement and also indicate that even traces amount of Al, Ti and Fe can be determined with good accuracy when using our method.

**Table 2:** Analytical results for silica sand – 1

Constituents	Set-I, % w/w, Results obtained using the proposed method	Set-II, % w/w Results obtained by complexometry^5 ^for Al, and colorimetry^6 ^for Fe and Ti
Al_2_O_3_	0.22 ± 0.03	0.23 ± 0.03
Fe_2_O_3_	0.18 ± 0.02	0.19 ± 0.03
TiO_2_	0.17 ± 0.02	0.19 ± 0.02

**Table 3:** Analytical results for silica sand – 2

Constituents	Set-I, % w/w, Results obtained using the proposed method	Set-II, % w/w Results obtained by complexometry^5 ^for Al, and colorimetry^6 ^for Fe and Ti
Al_2_O_3_	3.49 ± 0.11	3.45 ± 0.09
Fe_2_O_3_	0.33 ± 0.05	0.35 ± 0.04
TiO_2_	0.38 ± 0.05	0.40 ± 0.04

**Table 4:** Analytical results for silica Sand – 3

Constituents	Set-I, % w/w, Results obtained using the proposed method	Set-II, % w/w Results obtained by complexometry^5 ^for Al, and colorimetry^6 ^for Fe and Ti
Al_2_O_3_	9.92 ± 0.21	9.94 ± 0.24
Fe_2_O_3_	1.01 ± 0.10	1.00 ± 0.09
TiO_2_	0.38 ± 0.05	0.40 ± 0.05

**Table 5:** Analytical results for silica Sand – 4

Constituents	Set-I, % w/w, Results obtained using the proposed method	Set-II, % w/w Results obtained by complexometry^5 ^for Al, and colorimetry^6 ^for Fe and Ti
Al_2_O_3_	0.82 ± 0.08	0.86 ± 0.09
Fe_2_O_3_	0.98 ± 0.09	1.01 ± 0.1
TiO_2_	0.38 ± 0.05	0.43 ± 0.06

**Table 6:** Analytical results for foundry Sand – 5

Constituents	Set-I, % w/w, Results obtained using the proposed method	Set-II, % w/w Results obtained by complexometry^5 ^for Al, and colorimetry^6 ^for Fe and Ti
Al_2_O_3_	4.70 ± 0.14	4.72 ± 0.12
Fe_2_O_3_	0.94 ± 0.10	0.96 ± 0.09
TiO_2_	0.15 ± 0.05	0.19 ± 0.04

**Table 7:** Results of an 'accuracy check' of the proposed method with standard reference material: NIST SRM 81a Glass Sand

Constituents	Certified Reference Results as per certificate	Set-I, % w/w, Results obtained using the proposed method	Set-II, % w/w Results obtained by complexometry^5 ^for Al, and colorimetry^6 ^for Fe and Ti
Al_2_O_3_	0.66	0.67 ± 0.09	0.66 ± 0.10
Fe_2_O_3_	0.082	0.07 ± 0.02	0.08 ± 0.02
TiO_2_	0.12	0.15 ± 0.04	0.11 ± 0.04

**Table 8:** Results of an 'accuracy check' of the proposed method with standard reference material: NIST SRM 89 Lead Barium Glass

Constituents	Certified Reference results	Set-I, % w/w, Results obtained using the proposed method	Set-II, % w/w Results obtained by complexometry^5 ^for Al, and colorimetry^6 ^for Fe and Ti
Al_2_O_3_	0.18	0.18 ± 0.03	0.054 ± 0.05
Fe_2_O_3_	0.049	0.036 ± 0.004	0.044 ± 0.005
TiO_2_	0.01	0.024 ± 0.006	0.026 ± 0.006

**Table 9:** Results of an 'accuracy check' of the proposed method with standard reference material: IPT 61 Glass Sand

Constituents	Certified Reference Results	Set-I, % w/w, Results obtained using the proposed method	Set-II, % w/w Results obtained by complexometry^5 ^for Al, and colorimetry^6 ^for Fe and Ti
Al_2_O_3_	0.054	0.048 ± 0.005	0.05 ± 0.004
Fe_2_O_3_	0.014	0.016 ± 0.003	0.021 ± 0.004
TiO_2_	0.026	0.030 ± 0.004	0.034 ± 0.003

## 3. Conclusion

The chemical, optical, mechanical properties of glass, ceramics and others allied products are largely dependent upon the quality of silica sand. Therefore a suitable analytical method is essential for the precise determination of Al, Fe and Ti in such materials. The method outlined in this study is selective and precise in determining the quantities of Al and moderate to low quantities of Ti and Fe (concentration range ≤6% to ≥0.015%) for routine analysis of silica sand and allied materials. The method is therefore very simple, rapid, inexpensive and less time consuming in comparison to other existing methods.

## 4. Experimental

### 4.1 Apparatus

Calibrated pipettes and volumetric flasks supplied by Borosil Glass Works Ltd. India were used. A platinum dish of 99.99% purity obtained from Arora Matthey (Kolkata) India, (associate of Johanson Matthey U.K.) was used after cleaning with potassium hydrogen sulphate (KHSO_4_) fusion and subsequent washing with de-ionized water before each experiment. The digestion process was carried out on a Laminar flow bench equipped with an appropriate ventilation system.

### 4.2 Reagents

Hydrochloric acid 12N, nitric acid 8N, 98% sulphuric acid, hydrogen peroxide, ammonia, perchloric acid, sodium acetate and hydrofluoric acid GR grade supplied by E. Merck (Germany) were used. De-ionized water (18 mega ohm resistivity) prepared from the Millipore milli- Q water purification system, USA, was used throughout.

### 4.3 Standard Solution

#### 4.3.1 EDTA solution, 0.01M

3.744 g of the disodium salt of EDTA were dissolved in deionised (DI) water and diluted to 1 L. The stock solution was standardized with standard 0.01 M zinc acetate solution using sodium acetate-acetic acid buffer (NaOAC-HOAC, pH 5.3) and xylenol orange as indicator.

#### 4.3.2 Lead nitrate Solution, 0.01M

3.312 g of lead nitrate were dissolved in DI water and acidified with a few drops of nitric acid before finally being diluted to 1 L with DI water. The stock solution was standardized with EDTA solution in NaOAC-HOAC buffer (pH 5.3) using xylenol orange as the indicator.

#### 4.3.3 Iron(III) ammonium sulfate solution, 0.01M

0.4911 g of ferrous ammonium sulfate were dissolved with DI water, 10 mL of sulfuric acid (1:1) and 5 mL of hydrogen peroxide. The whole solution was boiled for 15 minutes to decompose excess hydrogen peroxide, before being cooled and then diluted to 1 L with DI water. The stock solution was standardized against a standard EDTA solution at pH 2–3 using sulphosalicylic acid as indicator

#### 4.3.4 Standard titanium solution, 0.025M

0.5 g of titanium dioxide in a Pt-crucible were fused with 5 g potassium bisulfate. The cooled melt was dissolved in 25 mL of sulfuric acid and 200 mL DI water over a steam bath. The solution was then diluted to 500 mL and standardized with lead nitrate using a pH 5.3 buffer in the presence of hydrogen peroxide by back titrating excess EDTA using xylenol orange indicator.

#### 4.3.5 Standard aluminium solution, 0.025M

0.6745 g of polished aluminum foil were cleaned with absolute alcohol and then dissolved in 25 mL hydrochloric acid and 150 mL of DI water, before being further diluted to 500 mL. The stock solution was standardized by back titration of the excess EDTA at pH 5.3 using standard lead nitrate solution and xylenol orange indicator.

#### 4.3.6 Sodium acetate-acetic acid buffer (pH 5.3)

The 5.3 pH buffer solution was prepared by dissolving 21.5 g of sodium acetate and 2 mL of acetic acid in 300 mL, before being diluted up to 1 litre with DI water.

#### 4.3.7 Tartarate Solution

10 g of tartaric acid were dissolved in 500 mL DI water, before dilution to 1 L.

#### 4.3.8 Orthophenanthrolin solution 0.1% (w/v) in water

0.5 g of chromotropic acid were dissolved in 100 mL of 0.3 N sulfuric acid, before being stored in a dark coloured bottle. This solution was used for the determination of iron by spcetrophotometry.

#### 4.3.9 Ascorbic acid solution 10 % (w/v)

10.0 g of the ascorbic solution were dissolved in 100 mL DI water.

#### 4.3.10 Xylenol orange solution, 0.1% (w/v)

0.1 g of the xylenol orange solution were prepared by dissolving 0.1 g of xylenol orange solution in water, before acidification with 2–3 drops of hydrochloric acid (1:4).

#### 4.3.11 Procedure

1.0 g of a well ground sample obtained after loss on ignition (100 ± 5°C) was put onto a cleaned platinum dish, moistened with a few drops of water, before adding 2 mL perchloric acid and 10 mL of 40% HF acid. The platinum dish containing whole components was evaporated to dryness on a hot plate and the process repeated several times to ensure the total evaporation of silica as SiF_4_. Finally the residue was dissolved in 5 mL of 12 N hydrochloric acid and DI water. The final solution was clear and no turbidity appeared on keeping all the samples for several days (except for sample number 3, where the residue was fused with potassium hydrogen sulfate, before the cooled melt was dissolved in hydrochloric acid and the final volume made up to 100 mL with DI water).

For the determination of Al, Ti and Fe quantities, 10 mL aliquots stock solutions of each sample were put into a 250 mL conical flask to which were added 25 mL of 0.01M EDTA for the formation of Al-EDTA, TiO-EDTA and Fe-EDTA complexes. To these solutions a few drops of xylenol orange indicator were added. Upon the solution becoming red in colour, a few drops of hydrochloric acid (3:1) were added whereupon the reddish colour turned yellow. A further 20 mL of sodium acetate-acetic acid buffer solution were added to achieve pH 5.3, before dilution to 100 mL with water. The whole solution was boiled for 5 min and then cooled to room temperature before being titrated with standard lead nitrate solution until the yellow colour changed to a sharp pinkish red. This first titre value corresponded to the excess unconsumed EDTA. After the end point the solution was boiled for a further 5 minutes following the addition of 20 mL tartaric acid solution. The liberated EDTA was titrated against 0.01 M lead nitrate solution under hot conditions. At the end point the yellow colour changed to a crimson red colour. This second titre value corresponded to the amount of Ti present. The solution was then cooled to room temperature followed by the addition of 1.0 g of NaF before being again boiled for 5 min. The solution was then cooled to room temperature and 5 mL of a pH 5.3 buffer solution added. This solution was then titrated with standard lead nitrate solution. At the end point the yellow colour changed to a crimson red colour. This third titre value corresponded to the quantity of Al present. Similarly a blank titration for 25 mL of 0.01 M EDTA was performed using standard lead nitrate solution, giving the total titre value. The difference between the total and the sum of the first, second and third gives the quantity of Fe present.
